# The diagnostic value of next-generation sequencing technology in sepsis

**DOI:** 10.3389/fcimb.2022.899508

**Published:** 2022-09-14

**Authors:** Xiao-guang Cao, Shu-sheng Zhou, Chun-yan Wang, Kui Jin, Hua-dong Meng

**Affiliations:** ^1^ Department of Emergency Medical Center, the First Affiliated Hospital of University of Science and Technology of China (Anhui Provincial Hospital), Hefei, China; ^2^ Department of Emergency Intensive Care Unit (EICU), the third Affiliated Hospital of Anhui Medical University, (the First People’s Hospital of Hefei), Hefei, China

**Keywords:** Next-generation sequencing, sepsis, pathogen, diagnosis, ICU

## Abstract

**Objective:**

This study aims to assess the clinical utility of next-generation sequencing (NGS) in sepsis diagnosis.

**Methods:**

A prospective study was conducted on patients with a high suspicion of sepsis by unknown pathogens from January 2017 to December 2021. Blood samples were taken from patients to perform NGS, blood culture (BC), leucocyte (WBC), procalcitonin (PCT), creatinine (CREA), Albumin (ALB) and C-reactive protein (CRP) tests.

**Results:**

The feedback time for BC was 3~5 days for bacteria and 5~7 days for fungi, while the turnover time for NGS was only 24 h. The clinical diagnosis was considered the “gold standard”. 83 patients passed our inclusion criteria and were separated into two groups by clinical diagnosis. 62 met the clinical diagnosis criteria for sepsis and 21 were non-sepsis. The data from the two groups were retrospectively compared and analyzed. Of 62 sepsis in 83 patients, 8(9.64%) were diagnosed by both BC and NGS, 51 (61.45%) by NGS only, 1(1.20%) by BC and 2 (2.41%) by conventional testing only; PCT, CREA, CRP levels and the detection rate of NGS and BC were higher in the sepsis group than in the non-sepsis group, while ALB levels were lower (p<0.05). The logistic regression results in our study revealed that NGS and ALB were independent prediction factors for sepsis (*p*<0.05), the area under the receiver operating characteristic curve (AUC), sensitivity and specificity of NGS for diagnosing sepsis was 0.857, 95.16% and 76.19%, while ALB was 0.728, 58.06%, 80.95%, respectively. The combination’s sensitivity, specificity and AUC of NGS and ALB were 93.55%, 85.71% and 0.935, greater than that of Albumin or NGS only (both *p*<0.05).

**Conclusion:**

NGS can effectively and quickly identify pathogens, thereby emerges as a promising technology for sepsis diagnosis. Combination of NGS and ALB can be used for early screening and is more powerful than NGS or ALB only.

## Introduction

Sepsis is a leading cause of intensive care unit (ICU) admission and death in ICU patients (Fleischmann-Struzek et al., 2020; Vidrine et al., 2020). It contributes to one-third to one-half of all hospital deaths and is responsible for over five million deaths worldwide each year (Sakr et al., 2018; Markwart et al., 2020; Rudd et al., 2020). Although the incidence has been steadily decreasing in recent years (Rudd et al., 2020), it remains a challenge for Chinese doctors (Xiaotong et al., 2017; Qu et al., 2021). Although sepsis guidelines are constantly updated (Levy et al., 2001; Hernández et al., 2016; Shankar-Hari et al., 2016; Seymour et al., 2016; Driessen et al., 2018), early pathogen identification and determination is the critical link between sepsis diagnosis and treatment (Rhee et al., 2020). Bacteria in the blood are responsible for most cases, but not all sepsis is caused by bacterial infections, and these infections can also be attributed to atypical pathogens, fungi, viruses, and protozoa (Sinha et al., 2018).

Nonetheless, pathogen identification is primarily based on culture methods, molecular methods, and so on, which are unsatisfactory in specificity, sensitivity, and time. Currently, blood culture is currently the most commonly used method for diagnosing sepsis and bloodstream infections (Rhodes et al., 2017). Its clinical applicability is limited due to its insensitivity and long culture cycle. In this context, culture-independent molecular diagnostic approaches (e.g., polymerase chain reaction (PCR)-based techniques and antibody detection technology) have already been used in patients to identify the causative pathogen (Marco, 2017). However, the occurrence of ambiguous results and limitations in pathogen pre-judgment are known limitations of these approaches. As a result, an effective and quick method to determine the presence of pathogens and infections is critical for patient triage in ICU setting (Dubourg et al., 2018; Sinha et al., 2018; Richter et al., 2019).

NGS has gained popularity in recent years due to its high sensitivity for pathogen detection. Compared to the traditional cultural method, NGS, an emerging innovative technology, has advanced infection diagnosis (Richter et al., 2019; Wilson et al., 2019; Fang et al., 2020). It is a powerful tool with benefits such as full-spectrum detection, a short detection cycle, and semi-quantitative analysis. However, the explanation of NGS results is complicated and lacks clear criteria, which has been bothering clinicians for a long time. Moreover, there lacks large scale multicenter prospective study even until recent years. As a result, from January 2017 to December 2021, a study was conducted on patients with a high suspicion of sepsis. This study aims to examine the applicability of NGS for pathogen detection in plasma to provide a novel approach to diagnosis and treatment of patients with sepsis in ICU.

## Materials and methods

### Data collection

**Figure 1:**
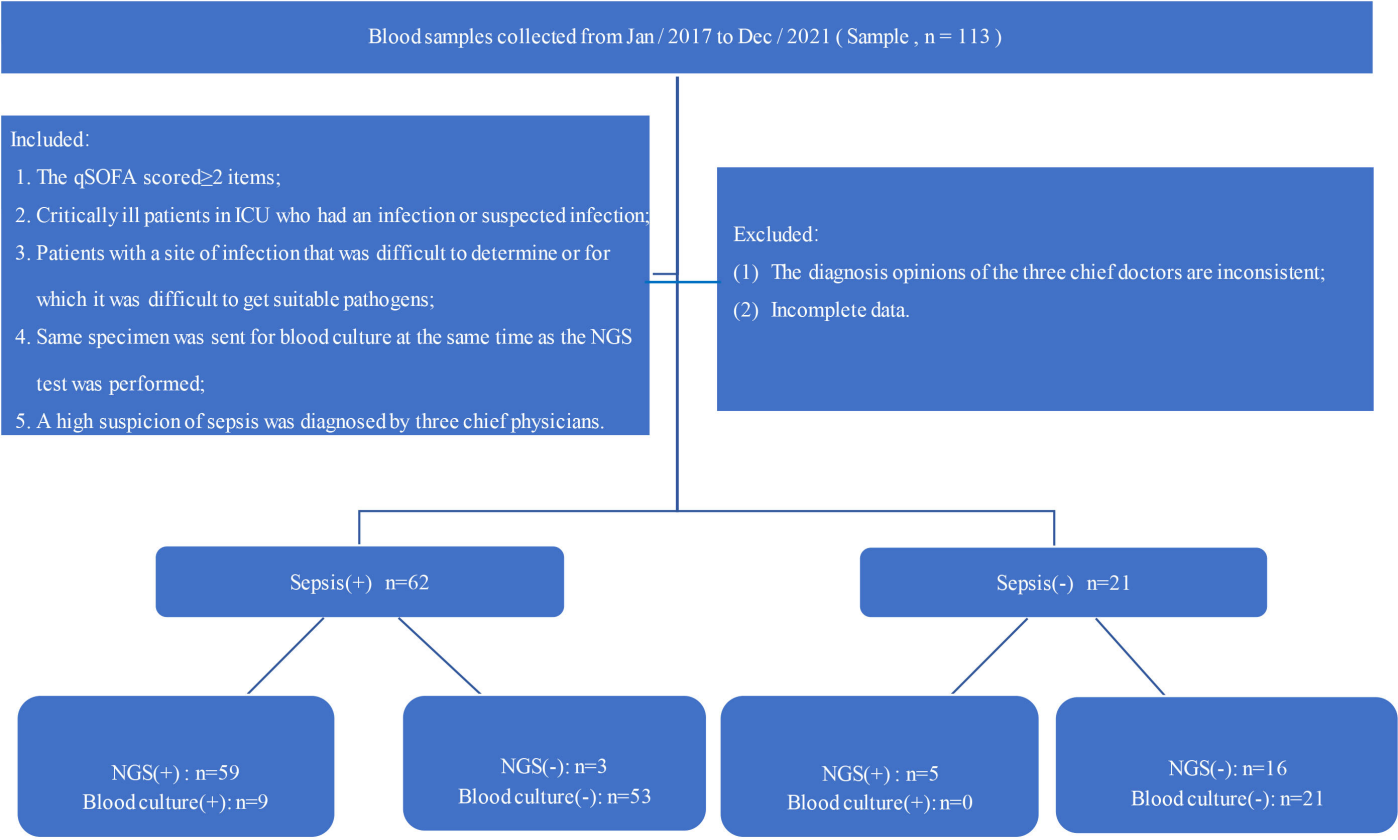
Flow chart of the study; qSOFA: blood pressure ≤100 mmHg, respiratory rate ≥22 breaths per minute, Glasgow coma scale <15.

Between January 2017 and December 2021, a total of 113 patients suspected infection were screened and referred across three branch hospitals participating sites for review in this study (**Figure 1**). The following inclusion criteria were used: 1). The q SOFA scored≥2 items; 2). Critically ill patients in ICU who had an infection or suspected infection; 3). Patients with a site of infection that was difficult to determine or for which it was difficult to get accurate pathogens;4). Same specimen was sent for BC at the same time as the NGS test was performed; 5). A high suspicion of sepsis was diagnosed by three chief physicians. The following exclusion criteria were used: (1). The diagnosis opinions of the three chief doctors are inconsistent;(2). Incomplete data. All patients’ data were independently compiled by two doctors and proofread by a chief physician. Age, sex, WBC, CRP, PCT, ALB, CREA, blood culture (BC), and other indicators were collected from the Donghua electronic system. Meanwhile, since there is also a small amount of microbial cell-free DNA in the blood of healthy people, 16 healthy volunteers were chosen as negative controls [only one people was detected EBV virus(1/16),and the other results were negative(15/16)]. Furthermore, the data collected from patients are anonymized and encrypted to protect the patients’ privacy.

## NGS methodology

### Specimen collection and preparation

Volume of 5 mL of blood were drawn from patients, placed in acid-free nucleic tube and stored at room temperature for 3~5 minutes before plasma separation and centrifuged at 4,000 rpm for 10 min at 4°C within 8 h of collection. Plasma samples were transferred to new sterile tubes. About 200ng of DNA was extracted from 300uL of plasma using the TIANamp Micro DNA Kit (DP316, TIANGEN BIOTECH, Beijing, China) following the manufacturer’s operational manual. The extracted DNA specimens were used for the construction of DNA libraries.

### Construction of DNA libraries and sequencing

The extracted DNA was sonicated, yielding 200-300bp DNA fragments. Then, DNA libraries were constructed through DNA-fragmentation, end-repair, adapter ligation, and PCR amplification. Agilent 2100 was used for quality control of the DNA libraries. Qualified libraries were pooled, and DNA nanoballs (DNBs) were constructed using single-stranded DNA circles and sequenced o by BGISEQ-50/MGISEQ-2000 platform.

### Bioinformatic analysis

To generate high-quality sequencing data, low-quality reads were removed, followed by computational subtraction of human host sequences mapped to the human reference genome (hg19) using Burrows-Wheeler Alignment. The remaining data by removal of low-complexity reads were classified by simultaneously aligning to Pathogens metagenomics Database (PMDB), consisting of bacteria, fungi, viruses and parasites. The classification reference databases were downloaded from NCBI (ftp://ftp.ncbi.nlm.nih.gov/genomes/). Ref Seq contains2,328 bacteria, 199 fungi, 4,189 viruses, 135 parasites, 83 mycobacteria, and 41 mycoplasma/Chlamydia linked to human diseases.

### Criteria for a positive NGS result

If any of the following three items were met, the result was judged to be positive (Long et al., 2016; Consensus Group Of Experts On Application Of Metagenomic Next Generation Sequencing In The Pathogen Diagnosis In Clinical Moderate And Severe Infections et al., 2020).

(1). Bacteria (mycobacteria excluded), virus and parasites: NGS identified a microbe (species level) whose coverage rate scored 10-fold greater than that of any other microbes of the same type.
(2). Fungi: NGS identified a microbe (species level) whose coverage rate scored 5-fold higher than that of any other fungus because of its low biomass in DNA extraction.
(3). Mycobacteria: Mycobacterium tuberculosis (MTB) was considered positive when at least 1 read was mapped to either the species or genus level due to the difficulty of DNA extraction and low possibility for contamination. Nontuberculous mycobacteria (NTM) were defined as positive when the mapping read number (genus or species level) was in the top 10 in the bacteria list due to the balance of hospital-to-laboratory environmental contamination and low yield rate.
(4). The result complied with the clinician’s judgment based on clinical symptoms, laboratory tests, Chinese expert consensus (Consensus Group Of Experts On Application Of Metagenomic Next Generation Sequencing In The Pathogen Diagnosis In Clinical Moderate And Severe Infections et al., 2020).

## Sepsis 3.0 definition and clinical diagnosis

Sepsis is a potentially fatal organ dysfunction caused by an abnormal host response to infection and > 2 points can identify organ dysfunction in the total SOFA score due to illness (Rahmel, 2018). Three chief physicians utilized clinical symptoms, laboratory test results, China’s expert consensus (Consensus Group Of Experts On Application Of Metagenomic Next Generation Sequencing In The Pathogen Diagnosis In Clinical Moderate And Severe Infections et al., 2020) and sepsis 3.0 to diagnose sepsis and pathogenic bacteria.

## Statistical analysis

Continuous variables with normal distribution were expressed as mean ± standard deviation (SD), whereas continuous data with non-normal distribution were expressed as *M* (*P*25 and *P*75) and categorical data were expressed as numbers (percentage). Variables were compared using the *t*-test, the non-parametric Mann–Whitney *U* test, or *χ^2^* test, as appropriate. We used clinical diagnosis as reference standard to evaluate the diagnostic efficacy of different indexes. The diagnostic accuracy of clinical features and laboratory characteristics was evaluated and compared by using the area under the curve (AUC) of receiver operator characteristics (ROC). The SPSS 22.0 software and Medcala 18.11.3 software were used for data analysis. *p*-values < 0.05 were considered statistically significant.

## Results

### Patients’ baseline characteristics

In this study, approximately 113 patients met the inclusion criteria, of which 83 were enrolled, including 51 males and 32 females aged 1~92 years. All patients were divided into two groups based on their clinical diagnosis: non-septic patients (25.30%, 21/83) and septic patients (74.70%, 62/83). Of 62 sepsis in 83 patients, 8(9.64%) were diagnosed by both BC and NGS, 51 (61.44%) by NGS only, 1(1.20%) by BC and 2 (2.41%) by conventional testing only (**Table 1**). The levels of CREA, PCT and CRP in sepsis groups were higher than those of the non-sepsis group, while the level of ALB was lower (*p*<0.05). No significant differences were observed between the two groups regarding age, gender, WBC and the detection rate of blood culture detection(*p*>0.05) (**Table 1**).

**Table 1:** Comparison of demographic and diagnostic characteristics between two groups [n(%)].

Variables	Sepsis		*χ^2^*/*t/Z-*value	*p-*value
	Yes ( *n *= 62 )	No ( *n *= 21 )		
**Sex (M/F)**	40 (60.52)/22 (35.48)	11(52.38)/10 (47.62)	0.975	0.323
**Age (years)**	57.00 (47.75, 70.00)	52.00 (39.00, 67.00)	-0.660	0.509
**NGS (+/-)**	59 (95.16)/3 (4.84)	5 (23.81)/16 (76.19)	41.292	0.001<
**BC(+/-)**	9 (14.52)/53 (85.48)	0/21 (100)	–	0.103
**PCT**	2.05 (0.43, 8.62)	0.39 (0.23, 2.17)	-2.523	0.012
**A**LB	28.88 ± 5.55	33.39 ± 6.55	-3.423	0.001
**CREA**	131.14 (74.75, 208.80)	63.00 (39.33, 97.25)	-2.750	0.006
**WBC**	11.66 (6.48, 15.20)	11.39 (8.14, 22.59)	-0.217	0.828
**CRP**	122.01 (38.83, 252.56)	42.42 (11.85, 168.12)	-2.250	0.024

### Correlation and concordance between NGS and BC

**Figure 2:**
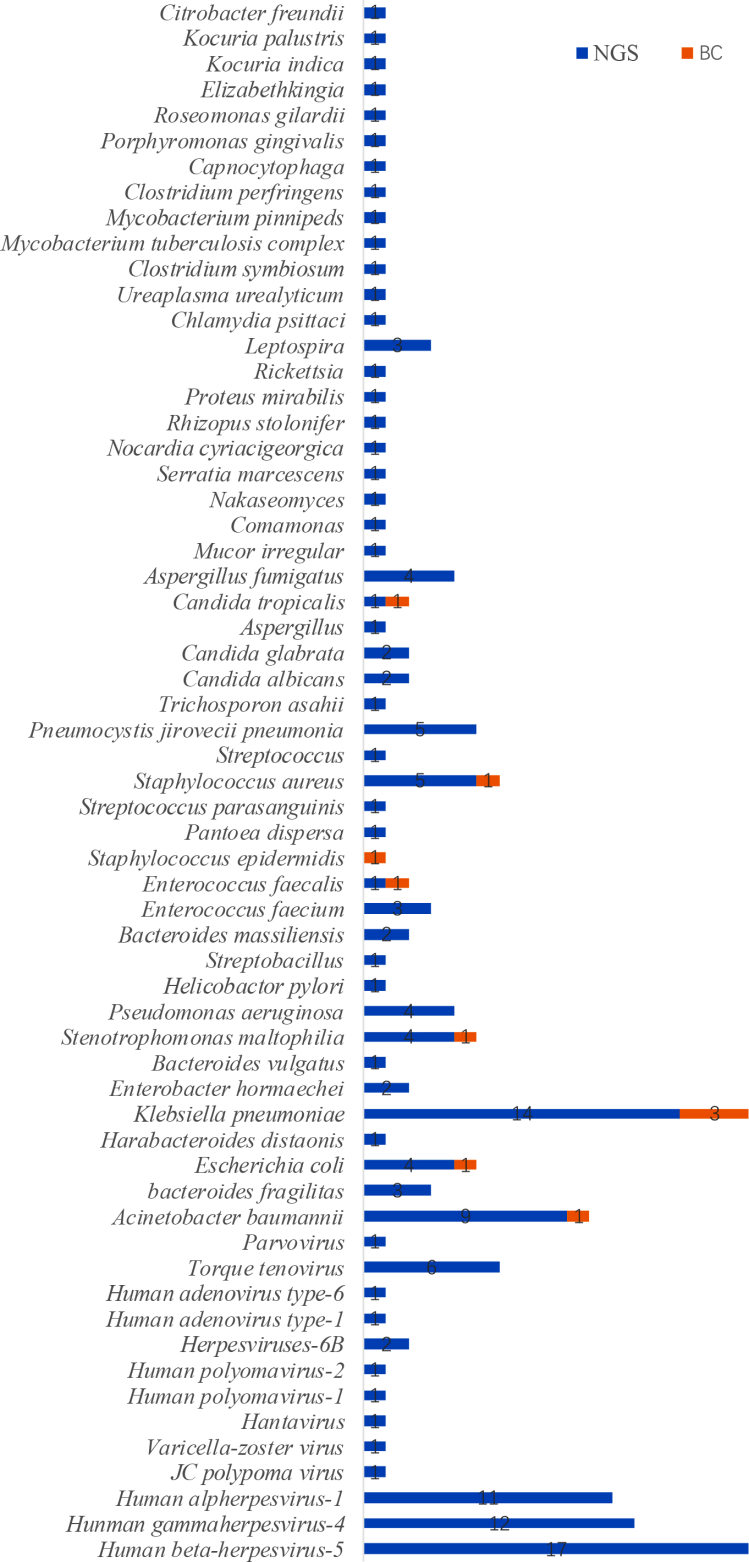
Distribution of pathogens identified by NGS and BC.

Given that patients may be colonized or infected by various pathogens in the complex environment of ICU, we counted all pathogens that met the positive criteria by NGS and BC. In total, NGS identified 59 septic patients, as compared with 9 septic patients with BC alone. 10 pathogens were identified in 9 blood samples(*Klebsiella pneumoniae* and *Candida tropicalis* were identified in one blood samples), including *Klebsiella pneumoniae* (3 cases), *Acinetobacter baumannii* (1 case), *Enterococcus faecalis* (1 case), *Staphylococcus epidermidis* (1 case), *Stenotrophomonas maltophilia* (1 case), *Staphylococcus aureus* (1 case), *Escherichia coli* (1 case), and *Candida tropicalis* (1 case).By NGS, 44 samples contained two or more pathogens (53.01%), *Klebsiella pneumoniae* (14 cases), *Acinetobacter baumannii*(9 cases), *human alpherpesvirus-1*(11 cases), *human gammaherpesvirus-4*(12 cases) and *human beta-herpesvirus-5* (17 cases) were the most common pathogens (**Figure 2**). In addition, NGS has the advantage of a shorter turnaround time. The average feedback time for blood culture was 3~5 days for bacteria and 5~7 days for fungi, while the turnover time for NGS was only 24 h.

### Multi-factor logical regression analysis

NGS and ALB were found to be the independent factors for sepsis in a multi-factor logistic regression analysis (**Table 2**).

**Table 2:** Logistic regression analysis of sepsis.

Variables	*B*	*Wald*	*p*-value	*OR*	*95% CI*
**NGS**	-5.658	15.750	0.001<	0.003	0.000~0.057
**ALB**	-0.182	4.166	0.041	0.833	0.699~0.993
**CREA**	0.002	0.896	0.344	1.002	0.998~1.007
**PCT**	0.126	0.836	0.360	1.134	0.866~1.484
**CRP**	0.007	1.037	0.309	1.007	0.994~1.019

### Analysis of diagnostic-effectiveness of different indexes for sepsis

**Figure 3:**
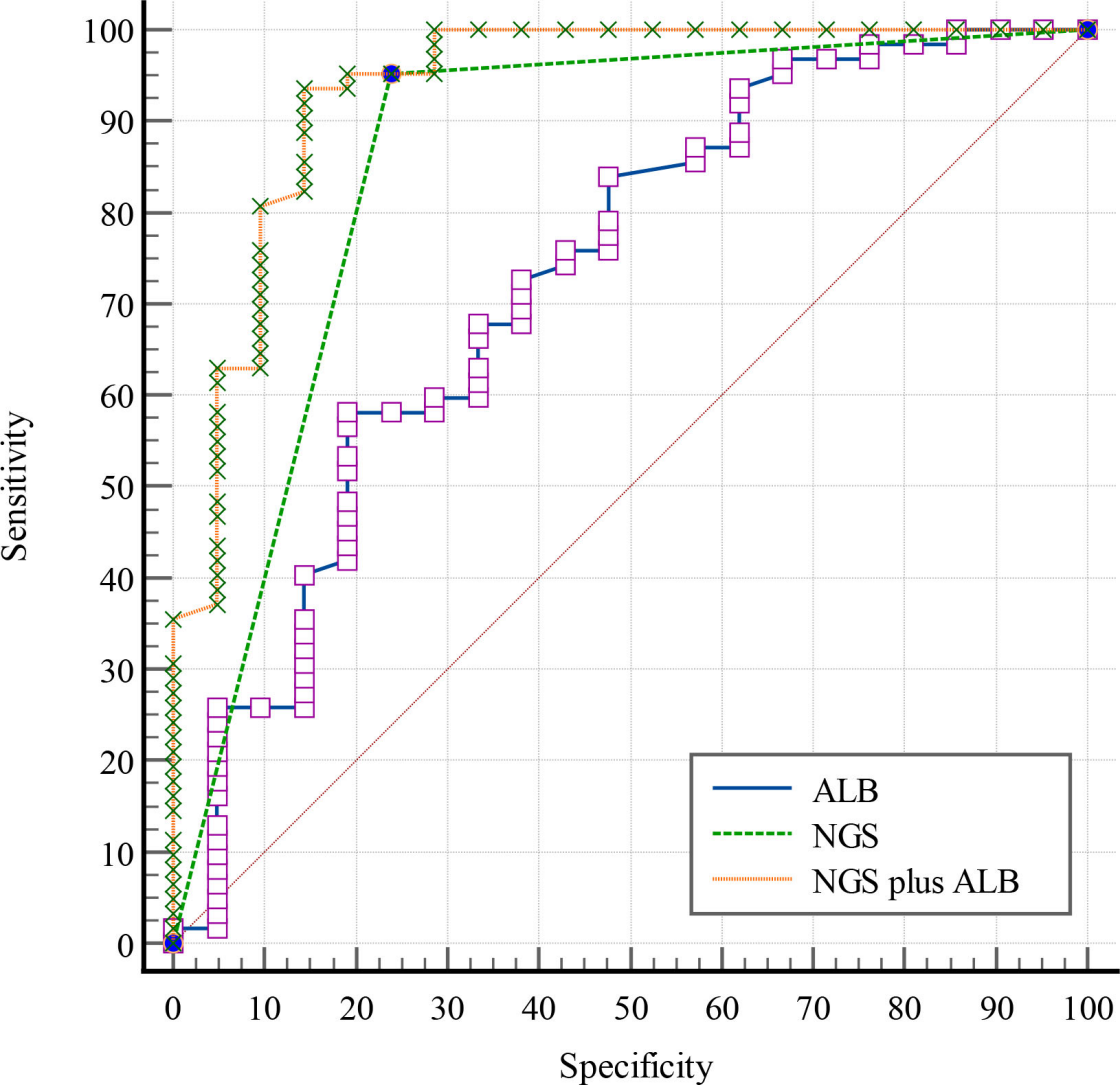
ROC curves of different indexes.

Using clinical sepsis diagnosis as the “gold standard.” ALB had an area under ROC curve of 0.728 (95% *CI*: 0.6190~0.820), 58.06% sensitivity and 80.95% specificity (optimal cutoff value:30.2g/L), while NGS had an area under the ROC curve of 0.857 (95% *CI:*0.763~0.924),95.16% and 76.19%. Meanwhile, the negative and positive prediction value of NGS were 84.21% and 92.19%, respectively. No significant differences in AUC was observed between ALB and NGS (Z=1.444, *p*=0.149, 95%*CI*:-0.046~0.304).The sensitivity and specificity of NGS plus Alb were 93.55% and 85.71%, respectively, while AUC [0.935 (95% *CI*: 0.859~0.978)] was higher than individually (NGS plus ALB vs NGS:*Z*=2.217, *p*=0.027, 95%*CI*:0.009~0.148; NGS plus ALB vs ALB: *Z*=3.023, *p*=0.003, 95% *CI*:0.073~0.342) (**Figure 3**).

## Discussion

Positive blood cultures are obtained in only a fraction of patients suffering from sepsis or septic shock, despite a proven underlying bacterial infection of 33% (Ransom et al., 2021). This is partly due to technical shortcomings in blood culture acquisition and local foci, fastidious organisms, or meagre rates of viable microorganisms in the bloodstream. Sepsis is a nasty infection in the intensive care unit that can result in septic shock or even death (Fang et al., 2020; Rudd et al., 2020 ). Separate international studies found that rapid and early identification of sepsis and pathogens is the most important prognostic factor, but the results were varied and complex (Perner et al., 2016; Campion and Scully, 2018). Clinical doctors’ preliminary judgment of sepsis is based primarily on inflammatory markers and etiological detection, such as blood culture, CRP, PCT, WBC, and other definitive tests. The accuracy and rapidity of pathogens disease judgment is the most concerning problem for doctors in ICU, directly related to treatment selection and patient prognosis.

CRP and PCT are commonly used and less expensive clinical detection methods directly linked to infection severity and is found to be increased by infections (Pontrelli et al., 2017; Cui et al., 2019; Tan et al., 2019; Friedman et al., 2021; Martin et al., 2021). A PCT of 2 ng/mL is usually indicative of sepsis. When a severe infection is caused by an inflammatory disorder caused by pathogen infection, particularly negative bacilli, serum PCT and CRP levels rise rapidly. As a result, PCT and CRP can be used to diagnose sepsis as a preliminary infection index. Furthermore, a progressive increase in CRP and PCT values can indicate an active state of infection, with values exceeding 100-fold higher than baseline. They can more accurately reflect the degree of inflammatory action than WBC, temperature, and other indices. In addition, the results are available about 1 h after admission. Many studies have suggested that CREA, CRP, and PCT may help diagnose sepsis in ICU. This finding was also confirmed by Zhang et al. (Cui et al., 2019). In our study, PCT and CRP levels were higher in the sepsis group than in the non-sepsis group, consistent with previous research (Cui et al., 2019; Martin et al., 2021). Under non-infection conditions, PCT and CRP levels are within normal ranges and are maintained in patients at a relative equilibrium. Meanwhile, a systemic inflammatory response can cause not only an increase in CRP and PCT but also renal dysfunction. If a patient develops sepsis shock, it can cause insufficient renal perfusion and worsen renal damage (Gómez and Kellum, 2016; Perner et al., 2016; Rahmel, 2018; Feng et al., 2021; Prescott and Angus, 2018).and our research also supported this conclusion. However, as a non-specific index, CREA, CRP, and PCT can be influenced by various factors, as evidenced by studies (Tan et al., 2019). The logistic regression results in our study revealed that PCT, CRP, and CREA were not independent risk factors for sepsis (*p*>0.05), consistent with the findings of numerous studies. Due to the confounding factors, the significance of being an independent predictor of sepsis requires further research.

Furthermore, ALB’s role as a mediator of proinflammatory molecules and inflammation should be given more attention (Zhang et al., 2003). Many researchers pay close attention to the relationship between sepsis and ALB (Das, 2015; Pimienta et al., 2019). There was a strong correlation between protein and inflammatory mediators (Pimienta et al., 2019). In patients with hypoproteinemia, plasma concentrations of IL-10, lipoxins, resolvins and protectins were significantly lower (Zhang et al., 2003; Das, 2015; Pimienta et al., 2019). Meanwhile, increased vascular permeability induced an ALB distribution alternation between intravascular and extravascular compartments (Wiedermann, 2021). As a result, hypoproteinemia is a common occurrence in patients with sepsis and is a strong predictor of morbidity (Zhang et al., 2003; Wiedermann, 2021). This study revealed that ALB levels were lower in the sepsis group than in the non-sepsis group, and logistic regression demonstrated that decreased albumin level is associated with increased risk for sepsis. This finding indicated that protein levels were negatively correlated with sepsis. As a result, decreased ALB concentrations were indicative of sepsis. Furthermore, hypoalbuminemia is associated with the diagnosis and prognosis of sepsis, which increases mortality rates during ICU admission. As a result, if sepsis is suspected, Albumin levels should be monitored at the time of entry.

Compared with the previous NGS technology (Long et al., 2016), it has been more mature, steadily improving and becoming more widely used in clinical practice in recent years, providing a powerful tool with advantages such as short turnaround time (24 h), wide microbial spectrum and semi-quantitative analysis (Greninger and Naccache, 2019; Linoj, 2019). Because it can directly detect thousands of fragments in a single test, this technology can reduce the number of tests and detection time (Linoj, 2019). It is less affected by antibiotics and pathogen activity. As a result, it has apparent advantages in pathogen identification. In contrast, blood culture’s long turnaround time and low sensitivity practically limit its clinical application, even though blood culture is the gold standard. The benefits of NGS are evident in this study: NGS reported 64 cases of positive samples, with a detection rate of 77.11% (64/83), whereas blood culture had a detection rate of only 10.84% (9/83), significantly lower than the reported level (about 30%) (Rhodes et al., 2017). Two possible causes for this were that many patients received broad-spectrum antibiotics before sampling during intra- or out-of-hospital (Davidson et al., 2019; Timsit et al., 2020).and the blood samples were collected at an inconvenient time or with insufficient blood volume (Henning et al., 2019). This finding also demonstrated that DNA of pathogenic microorganisms is retained in patient plasma for a more extended period too (Geng et al., 2021). Moreover, NGS has the advantage of a shorter turnaround time. The average feedback time for blood culture was 3-5 days for bacteria and 5-7 days for fungi, while the turnover time for NGS was only 24 h.

Previously, clinical doctors hypothesized that the low detection rate of blood culture was due to problematic pathogens to culture and had a long turnaround time, such as mycoplasma, chlamydia, and viruses (Opota et al., 2015). However, NGS results in this study revealed that these microorganisms accounted for an important part of ICU pathogens, such as human beta-herpesvirus-5, human alpherpesvirus-1 and human gamma herpesvirus-4. In addition, NGS identified pathogen species and diagnosed numerous mixed infections. NGS detected more than two pathogens in the blood samples of 44 patients (53.01%), whereas blood culture detected only one patient (1.20%). In comparison, blood culture typically results in identifying a single pathogen, which could be attributed to competition between pathogen types and a demanding growth environment (Jing et al., 2018).

According recent studies (Voiriot et al., 2016; Southeast Asia Infectious Disease Clinical Research Network, 2017; Liu et al., 2020), viruses should play an important role in sepsis. However, viruses were not considered as an important causative agent of sepsis in previous study (Long et al., 2016). NGS identified more than ten different types of viruses in this study. The diagnosis of viral infection is obvious limited by traditional methods. Even though molecular assays, such as polymerase chain reaction (PCR)-based methods, have been established for clinical detection, the detection spectrum is limited to known pathogens listed on the panel. Because few patients use traditional methods and incomplete date, this study did not statistically analyze this result. Although doctors did not absolutely confirm that these viruses were the single cause of sepsis after examining the patients’ conditions, 16 patients received anti-virus treatment. A paper found that patients with viral-bacterial co-infections were more seriously ill than those with only bacterial infections, including a higher frequency of hemodynamic disturbances, respiratory failure, and hospitalization (Voiriot et al., 2016). Hence, viruses may play a more important role than bacterium in some septic patients. As a result, we believe that detecting viruses is an essential supplementary means of determining the best treatment in ICU.

The study’s findings revealed that AUC of NGS was 0.857, with 95.16% sensitivity and 76.19% specificity; thus, NGS can be used as an essential supplementary means when pathogen acquisition is complex in ICU. Due to low specificity, NGS reports must still be carefully interpreted to avoid antibiotic abuse. The criteria for positive NGS results used in this research were those described by Chinese expert consensus and Long et al. (Long et al., 2016; Consensus Group Of Experts On Application Of Metagenomic Next Generation Sequencing In The Pathogen Diagnosis In Clinical Moderate And Severe Infections et al., 2020). Therefore, the criteria cannot finally definitively determine which microbes are pathogenic, background microorganisms and the detection of circulating cell-free DNA from non-pathogenic microbes. Thus, the fourth item in the list of criteria is especially important. Fortunately, in our study, NGS typically detected one or two types of pathogens, with no more than six pathogens found in a single blood sample. Although ALB and NGS alone were insufficient to diagnose sepsis, diagnostic efficiency was significantly improved when combined (*p*<0.05). The AUC of NGS plus ALB was 0.935, with a sensitivity of 93.55% and a specificity of 85.17%, indicating that it can be used as an essential supplementary means to reduce the likelihood of missed diagnosis and misdiagnosis.

Overall, sepsis is a series of clinical syndromes caused by the immune response to infection, and understanding the root of sepsis may help to facilitate identification of high-risk patients and prevent further deterioration. For patients who developed sepsis, the application of the NGS method for blood sample will improve the sensitivity of pathogen detection, notably for rare pathogens and fastidious bacteria. Combining NGS with ALB can further improve the sensitivity and specificity of diagnosis. Moreover, NGS is a promising test for the detection of mixed infections. However, the interpretation of NGS results must be associated with laboratory examination and clinical physical examination due to the lack of unified diagnostic criteria, especially for septic patients, which is also the core of this study. In conclusion, the advances in diagnosis approaches may help to develop better strategies for precise diagnosis and treatment of sepsis in the future.

### Limitations

This study has limitations as follows: first, in our results, all patients were from ICU, while the majority of samples were derived from blood, which may lead to biased conclusions if generalizing to a broader scale of clinical infectious disease. Second, few recognized criteria for sequencing result explanation have been established (interpretation of results, database of organism genome sequences, etc). So, in the practical application of the technology, the subjective judgment of clinicians is still needed, which is greatly influenced by clinical experience. Third, while results were reached in this study by three chief doctors based on clinical manifestations of patients combined with other laboratory results, subjective bias remains unavoidable. Fourth, because of the high cost of NGS testing (US $560), most enrolled patients have better economic circumstances or non-restricting health insurance, which may lead to selection bias and have an uncertain impact on the research results. Finally, because this method is very sensitive, a strict aseptic operation is required during the detection process; otherwise, clinical misdiagnosis is possible. Furthermore, due to the small sample size of this single-center study, our findings require further validation in larger-scale clinical trials.

## Data availability statement

The datasets presented in this study can be found in online repositories. The names of the repository/repositories and accession number(s) can be found below: https://ngdc.cncb.ac.cn/gsa-human/; CRA007416.

## Ethics statement

Ethical review and approval were not required for this retrospective study on human participants in accordance with the local legislation and institutional requirements in China. Written informed consent was obtained from all subjects. Written informed consent was not distinguished from the individual(s) for the publication of any potentially identifiable data included in this research.

## Author contributions

X-gC and H-dM raised the idea and designed the study. X-gC conducted statistical analysis on the data. X-gC, H-dM, S-sZ wrote the manuscript. KJ and C-yW revised the paper. All authors read and approved the submitted version.

## Conflict of interest

The authors declare that the research was conducted in the absence of any commercial or financial relationships that could be construed as a potential conflict of interest.

## Publisher’s note

All claims expressed in this article are solely those of the authors and do not necessarily represent those of their affiliated organizations, or those of the publisher, the editors and the reviewers. Any product that may be evaluated in this article, or claim that may be made by its manufacturer, is not guaranteed or endorsed by the publisher.
